# Supplementary material for: Comparison of obstetric emergency clinical readiness: A cross-sectional analysis of hospitals in Amhara, Ethiopia
Source: PLoS One. 2023 Aug 3;18(8):e0289496. doi: 10.1371/journal.pone.0289496 (PMC10399735; doi:10.1371/journal.pone.0289496)
Supplement: S1 Table — (DOCX) [file pone.0289496.s001.docx]

**Supporting Information**

**S1 Table. Availability of Critical Obstetric Emergency Resources**

| **Category** | **Type** | **Specific Tracer Item** | **%** | **n** ^a^ |
| --- | --- | --- | --- | --- |
| **Consumables**  and **Durables** | Consumable Supplies | Gloves, Aseptic | 35% | 7 |
|  |  | IV Fluid ^b^ | 95% | 19 |
|  |  | IV Kit ^c^ | 100% | 20 |
|  | Durable Goods and Infrastructure | Manual Vacuum Aspirator (MVA) | 100% | 20 |
|  |  | Light source ^d^ | 80% | 16 |
| **Drugs** | Uterotonic: First Line | Parenteral oxytocin | 95% | 19 |
|  | Uterotonic: Alternatives | Oral misoprostol | 55% | 11 |
|  |  | Parenteral ergometrine | 80% | 16 |
|  |  | Oxytocin or misoprostol | 95% | 19 |
|  |  | Any uterotonic ^e^ | 95% | 19 |
|  | Antibiotic 1: First Line | Parenteral ampicillin | 95% | 19 |
|  | Antibiotic 1: Alternative | Any parenteral penicillin^f^ | 5% | 1 |
|  |  | Ampicillin or any parenteral penicillin | 95% | 19 |
|  | Antibiotic 2: First Line | Parenteral gentamicin | 35% | 7 |
|  | Anticonvulsant: First Line | Parenteral magnesium sulfate | 100% | 20 |
|  | Anticonvulsant: Alternative | Parenteral diazepam | 45% | 9 |
| **Protocols - Algorithms** | Medical Treatments | Hemorrhage | 35% | 7 |
|  |  | Eclampsia | 75% | 15 |
|  |  | Infection-Sepsis | 0% | 0 |
|  | Manual Procedures | Retained Placenta | 15% | 3 |
|  |  | Incomplete Abortion | 15% | 3 |
|  | General | Obstetric Emergency Manual | 60% | 12 |

^a^n=20 facilities

^b^Either normal saline (NS) or lactated ringer’s (LR)

^c^IV cannula

^d^functional electric lights and electricity or flashlights

^e^Presence of one or more of the following parenteral drugs: oxytocin, misoprostol, ergotomine

^f^Parenteral ampicillin or any parenteral penicillin alternative (benzathine, procaine or crystalline
