# Supplementary material for: Comparison of obstetric emergency clinical readiness: A cross-sectional analysis of hospitals in Amhara, Ethiopia
Source: PLoS One. 2023 Aug 3;18(8):e0289496. doi: 10.1371/journal.pone.0289496 (PMC10399735; doi:10.1371/journal.pone.0289496)
Supplement: S2 Table — (DOCX) [file pone.0289496.s002.docx]

**S2 Table.** **Comparison of Emergency Readiness Using Clinical Cascades and Signal Functions, Full Sample ^a,b^**

| **Clinical Cascade** | **Signal Functions** | **Clinical Cascades** | **Overestimated Readiness** |
| --- | --- | --- | --- |
| *(Signal Function)* | *% Readiness, Tracer Items* | *% Readiness, Stage 2* | *[Signal Function (-) Cascade]* |
| **Medical Treatments** | | | |
| **Manage Sepsis- Infection** *(Antibiotic)* | 91% | 50.0%  (n=10) | 41.0% |
| **Manage Hemorrhage** *(Oxytocic)* | 96% | 75.0%  (n=15) | 21.0% |
| **Manage Hypertensive Emergency** *(Anticonvulsant)* | 98.8% | 85.0%  (n=17) | 13.8% |
| *Medical Readiness, Pooled Mean* | **95.3%** | **70.0%** | **25.3%** |
| **Manual Procedures** | | | |
| **Manage Retained Placenta** *(Manual removal of retained placenta)* | 90.7% | 30.0%  (n=6) | 60.7% |
| **Manage Incomplete Abortion** *(Manual removal of retained products of conception)* | 90.7% | 65.0%  (n=13) | 25.7% |
| **Manage Prolonged Labor (***Assisted Vaginal Deliver)* | 90% | 75.0%  (n=15) | 15.0% |
| *Manual Readiness, Pooled Mean* | **90.5%** | **56.7%** | **33.8%** |
| **Overall Pooled Mean Readiness** | **92.9%** | **63.3%** | **29.6%** |
|  | *Signal Function Estimate* | *Cascade Estimate* | *% Overestimated Readiness by Signal Functions* |

^a^n=20 facilities

^b^The total number and percentage of facilities out of 20 that DO have the readiness to treat the specified emergency
