# Supplementary material for: Comparison of obstetric emergency clinical readiness: A cross-sectional analysis of hospitals in Amhara, Ethiopia
Source: PLoS One. 2023 Aug 3;18(8):e0289496. doi: 10.1371/journal.pone.0289496 (PMC10399735; doi:10.1371/journal.pone.0289496)
Supplement: Attachment — Submitted filename: Respone to Reviewers.docx [file pone.0289496.s003.docx]

To the reviewers of this manuscript,

I want to thank you for taking the time to review this work and providing useful critiques. Below is a table where our team responds to each of the comments presented by the reviewers individually. Again, our team appreciates your time and hope the edits will resolve any concerns related to this manuscript.

Best,

Kylie Dougherty

| **Reviewer** | **Author Response** |
| --- | --- |
| **Journal Requirements** |  |
| Please ensure that your manuscript meets PLOS ONE’s style requirements | The manuscript has been edited to meet PLOS ONE’s style requirements |
| Please include a complete copy of PLOS’ questionnaire on inclusivity in global research in your revised manuscript. | A completed version of the questionnaire has been uploaded as Supporting Information |
| Please state what role the funders took in the study. | We have updated our funding statement to describe the role each funder took in this study. The updated statement can be found in our cover letter. |
| Related to data availability: please address the following prompts: | We have uploaded a minimal anonymized data set to DANS data repository. The data set can be found at <https://easy.dans.knaw.nl/ui/datasets/id/easy-dataset:305060> |
| Please ensure that you include a title page within your main document. You should list all authors and all affiliations as per our author instructions and clearly indicate the corresponding author. | A title page has been included in the unmarked version of our revised paper labeled ‘Manuscript’ |
| Your ethics statement should only appear in the Methods section of your manuscript. If your ethics statement is written in any section besides the Methods, please delete it from any other section. | Our Ethics statement has been removed from the end of our manuscript and can only be found under the Methods section of our paper now. |
| Please include captions for your Supporting Information files at the end of your manuscript, and update any in-text captions to match accordingly. | A section following references has been added describing the information available in the supplemental material. |
| Please review your reference list to ensure that it is complete and correct. If you have cited papers that have been retracted please include the rationale for doing so in the manuscript text, or remove these references and replace them with relevant current references. | Upon reviewing our reference list we have not removed any citations from the previous version. We have included one new reference that can be found on pages 6-7 of the manuscript where we discuss SLL in greater detail. |
| **Reviewer #1 Comments** |  |
| This is a good study, pointing out key factors of obstetric emergency clinical readiness. | We appreciate your kind words and support for our work. |
| Editing of text- I realized your font varies in different sections | We have ensured the entire document is set to Times New Roman font |
| Be clear on the number of emergencies on focus, is it 5 or 6? On methods you mentioned 5, and on results you mentioned 6 | We have fixed this discrepancy and the manuscript only states six obstetric emergencies instead of five |
| On your key words, I think obstetric emergency should be added since it’s key in your title | We appreciate this recommendation and have included this additional key word |
| When starting a sentence, you put words i.e. line 5 of your introduction should not start with 94% but words | We have made the necessary correct to line 5 page 1. |
| Your list of abbreviations is incomplete- a number of abbreviations used in the document not included eg. CEmOC, BEmOC, SF, SRI etc. | The list of abbreviations has been updated to include all abbreviations referenced in the manuscript. |
| Page 18, line 7- reasonable request. Be clear what you mean by tomorrow that | We have uploaded a minimal anonymized data set to DANS data repository. The data set can be found at <https://easy.dans.knaw.nl/ui/datasets/id/easy-dataset:305060>, and the data availability statement will be updated by PLOSOne |
| **Reviewer #2 Comments** |  |
| I am very impressed by this research because it is tackling the top causes of maternal mortality in Sub-Saharan Africa…*strengths of the manuscript are mentioned* | We appreciate your kind words and support for our work. |
| Some abbreviations are missing on the list of abbreviations yet not easily understood such as: BEmOC, SRI, SLL, SF | The list of abbreviations has been updated to include all abbreviations referenced in the manuscript. |
| The manuscript needs to be proofread by English language expert for grammatical errors | We appreciate this recommendation, and the manuscript has been proofread by two native English speakers. |
| Line 5-8 on page 3 are confusing. It talks about dividing most common obstetric emergencies into 2 forms of emergencies which are in turn listed as readiness in lines 6 and 7 of page 3. Clarify emergencies vs. readiness | Thank you for pointing out this confusion. We have clarified definition of manual and medical readiness on page. |
| There are abbreviations used which have not been defined anywhere in the manuscript: EmOC on line 8 of page 6, CEmOC on line 16 of page 10, IRB on line 11 of page 7, BEmONC on line 23 of page 7 and OBGYN on line 20 of page 10 | The abbreviations have all been updated within the manuscript to have the terms spelled out the first time they appear in the paper. |
| The IRB number should be included if available under ethics approval section. This is to avoid doubts on approval status of the research | Per our ethics statement in the methods section, our study was exempt from IRB oversight because it was considered a public health activity by Emory IRB and the need for informed consent was waived by Emory IRB committee because the work was viewed as a system-level public health activity. For this reason, there is not an IRB number. |
| **Major Issues** |  |
| The abstract does not have the findings of the second objective which was to quantify the relationship between SF and cascade estimates of readiness. A summary of major findings of each objective should appear under results in the abstract in the order of the objectives | We believe the relationship between the SF and CC estimates is described in the results section where we state, “The overall SF estimate for all six obstetric emergencies was 17.5% greater than the estimates using the CC.” This overestimate of readiness is an aspect of the comparison of findings between the two tools. |
| Statement in line 33 on page 1 stated “but regional health planners are unable to identify the need” should be removed. This study did not determine the ability of regional health planners to identify facilities unprepared to handle obstetric emergencies. The conclusion should come from your key findings. | We have clarified this statement in the conclusion of the abstract to display the potential issues that may arise from facility readiness discrepancies without explicitly stating that regional health planners would be unable to act. |
| Give some brief introduction (in a line or two) about SLL program mentioned in lines 20 and 21 under materials and methods. It is mentioned as a study in line 15 of page 7 under data collection. Is this study part of an intervention or not? Clarify this | We have provided additional details related to the SLL project to page 6 lines 20-23 and page 7 lines 1 and 2. |
| What are twenty year one hospitals under data collection section? If they are the 20 hospitals where SLL program was being implemented in the first year, make it easy to understand. How long did data collection take? This should also clarify line 15 on page 10 under results section. | We appreciate this comment related to the clarity of our sample description. We have provided more details related to the sample and SLL on pages 6 and 7 and have provided more details about the sample on page 10 lines 18 and 19 of the results section. The data collection period was between May 17-27, 2021. |
| “These missing items highlight the critical need for accurate readiness monitoring to ensure that facilities do not run out of crucial supplies” on line 11 and 12 on page 11 of results should go to discussion section. Results are first presented without discussing them. | Thank you for identifying this, we have removed the statement from the results section. |
| The results and data analysis section should be presented in the order of study objectives. There is no heading for “quantifying the relationship between SF and cascade estimates of readiness” under results section. The discrepancies between SF and cascade estimate readiness can be stated under relationship between SF and cascade estimates of readiness | Thank you for pointing out this discrepancy we have included the title “quantifying the relationship between SDF and CC estimates of readiness” below the SF and CC discrepancies information in the results section. |
| An upcoming innovation/innovation is mentioned in this study “electronic dashboard”. It is also important to state that this is a baseline study for the development of an innovation. This is to guide the reader further on the future of this research and the innovation to follow. | We have provided additional information describing how this analysis is the first step in a larger project to track regional readiness in Amhara. The additional information can be found on page 17 lines 5-9. |
| How do your findings relate with already existing similar research in different parts of the world? This should be elaborated under discussion section | We have added the following information describing how our findings align with the current literature.  “Our findings align with previous research conducted in in Kenya and Uganda, which found SF tracer items overestimate facility readiness to manage obstetric emergencies.” |
| Author’s contribution section does not contain all the authors. Those who contributed to the work but do not meet the criteria for authorship can be mentioned in the Acknowledgements | We have revised the author’s contribution section to reflect the role all authors played in developing this manuscript. |
